# HIV and STI Prevalence among Female Sex Workers in Côte d'Ivoire: Why Targeted Prevention Programs Should Be Continued and Strengthened

**DOI:** 10.1371/journal.pone.0032627

**Published:** 2012-03-05

**Authors:** Bea Vuylsteke, Gisèle Semdé, Lazare Sika, Tania Crucitti, Virginie Ettiègne Traoré, Anne Buvé, Marie Laga

**Affiliations:** 1 Department of Public Health, Institute of Tropical Medicine, Antwerp, Belgium; 2 Global Program Management, Abidjan, Côte d'Ivoire; 3 Department of Population Research and Development, National Institute of Statistics and Applied Economy, Abidjan, Côte d'Ivoire; 4 Department of Clinical Sciences, Institute of Tropical Medicine, Antwerp, Belgium; 5 National Program for HIV Care and Treatment, Ministry of Health, Côte d'Ivoire; Vanderbilt University, United States of America

## Abstract

**Objective:**

To assess condom use and prevalence of STIs and HIV among female sex workers (FSWs), as part of a comprehensive monitoring and evaluation plan of a nationwide sex worker prevention project in Côte d'Ivoire.

**Design and Methods:**

Cross sectional surveys were conducted among FSWs attending five project clinics in Abidjan and San Pedro (2007), and in Yamoussoukro and Gagnoa (2009). A standardized questionnaire was administered in a face-to-face interview, which included questions on socio-demographic characteristics, sexual behaviour and condom use. After the interview, the participants were asked to provide samples for STI and HIV testing.

**Results:**

A total of 1110 FSWs participated in the surveys. There were large differences in socio-demographic and behavioural characteristics between FSW coming for the first time as compared to FSW coming on a routine visit. The prevalence of *N. gonorrhoeae* or *C.trachomatis* was 9.1%, 11.8% among first vs. 6.9% routine attendees (p = 0.004). The overall HIV prevalence was 26.6%, it was lower among first time attendees (17.5% as compared to 33.9% for routine attendees, p<0.001). The HIV prevalence among first attendees was also lower than the proportion of HIV positive tests from routine testing and counselling services in the same clinics.

**Conclusions:**

The results show a relatively high STI and HIV prevalence among FSWs in different cities in Côte d'Ivoire. In the light of these results, prevention efforts should continue to focus on FSWs in the country.

## Introduction

Sex workers and their clients are a critical group in the spread of HIV infection, in every region in the world [1]. Targeted interventions that aim to increase condom use and reduce transmission of Sexually Transmitted Infections (STIs) and HIV infection among sex workers and their clients have been shown to be feasible and effective [2]–[6].

Côte d'Ivoire is one of the countries most severely affected by the HIV/AIDS epidemic in West Africa, with a HIV prevalence in the adult population of 4.7% in 2005 [7].

In 1992 the Projet RETRO-CI of the Centers for Disease Control and Prevention of Atlanta (USA) in collaboration with the Ministry of Health of Côte d'Ivoire and the Institute of Tropical Medicine (ITM), Antwerp, Belgium, set up a first dedicated clinic for female sex workers (FSWs) in Abidjan, the “Clinique de Confiance”. A series of community and facility based surveys were conducted between 1991 and 1998 showing a substantial increase in condom use and a dramatic decline of HIV prevalence from 89% in 1992 to 33% in 1998 among sex workers attending the clinic for the first time [4].

**Figure 1:**
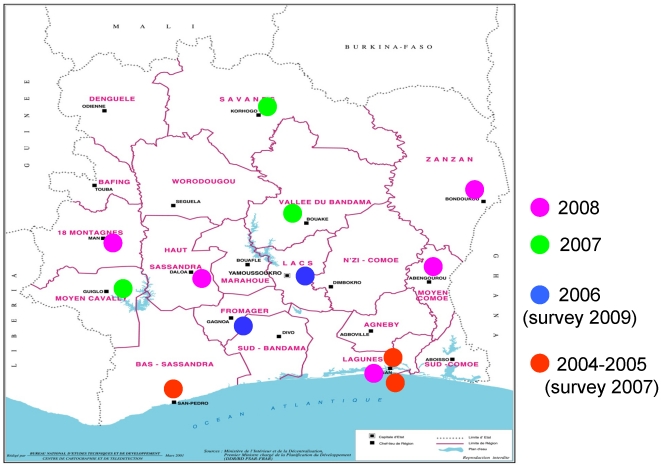
Scaling up sex worker interventions in Côte d'Ivoire: phased approach.

The success of this project called for expansion and scaling up of both community-based and clinic-based HIV prevention activities for FSWs in Côte d'Ivoire [4]. From 2004 onwards activities have been rolled out by Family Health International (FHI, now FHI360) and ITM with the financial support of the President's Emergency Plan for AIDS Relief (PEPFAR) and the Belgian Directorate-General for Development (DGD). The objectives of the project, which is named PAPO-HV (“Projet d'Assistance aux Populations Hautement Vulnérables”, or “Project for assistance to Highly Vulnerable Populations”), were to expand the reach and improve the quality of HIV prevention and care for FSWs in the whole country. The project used a phased approach with new clinic sites opening every year and by 2008, 13 sites in Côte d'Ivoire were providing services for FSWs (see figure 1). At each site, a comprehensive package of prevention and care services are offered, including behaviour change communication and condom promotion by and in collaboration with peer educators, STI screening and treatment, Counselling and Testing (CT) and care for the HIV infected. Key indicators of the project's success include an increase in condom use and a decrease of STI/HIV prevalence among the target population.

Since 2003 no data have been collected on STIs or HIV among FSWs in Côte d'Ivoire. An Aids Indicator Survey was conducted among the general population in 2005 in 2005, but no data has been collected on women offering sex for money or goods [7]. Conducting a nationwide bio-behavioural survey among sex workers was not an option during the PAPO-HV project because of logistics and most of all budget constraints. For this reason, a series of facility based surveys were planned, as part of a comprehensive monitoring and evaluation plan. The project evaluation team has prepared baseline surveys in a phased approach, to align with the stepwise opening of new project sites (see fig. 1). The first baseline surveys were conducted in 2007 and 2009 with the aim to assess condom use and prevalence of STIs and HIV. Further baselines surveys were not realised due to a change in funding in 2010.This paper reports on the results of these surveys in the five clinics and compares HIV prevalence with the proportion of HIV-positive FSW in routine counselling and testing services at the same clinics.

## Methods

### Design and setting

The cross sectional surveys were conducted in five of the 13 project clinics. Between October and November 2007, surveys were conducted in two clinics in Abidjan and in one clinic in San Pedro, which were all operational before 2004 (see figure 1). Between June and August 2009, surveys were conducted in Yamoussoukro and Gagnoa, where services for FSWs started in 2006. All clinics are run by local non-governmental organisations. Two clinics are dedicated to sex workers only (Clinique de Confiance in Abidjan and Centre Espérance in San Pedro), the other clinics are integrated in existing private not-for-profit health facilities.

### Study population

The study populations were FSWs of at least 15 years old. Sex workers were defined as women who provide sexual services in exchange for money, goods or other benefits. All consecutive female sex workers attending one of the study clinics were invited to participate, until the sample size of 220 participants per site was reached.

### Study procedures

Study procedures were identical in all surveys, with the exception of the HIV testing procedures and the STI treatment. A research assistant checked the eligibility of the potential study participants and asked for a written informed consent. A standardized questionnaire was administered in a face-to-face interview, which included questions on socio-demographic characteristics, sexual behaviour and condom use.

After the interview, the participants were asked to provide samples for STI testing by two self-administered vaginal swabs. They were also invited to provide samples for HIV testing. During the surveys conducted in 2007, an anonymous HIV test was performed on transudate of the gingival mucosa that was collected with a collection device from the manufacturer (OraSure Technologies Inc., Bethlehem, USA). Participants who wanted to know their HIV status were referred to the routine counselling and testing service where rapid tests were used on a blood sample. In the 2009 surveys study all study participants were invited to HIV counselling and testing services where the new national algorithm was applied using rapid tests on fingerprick blood. In other words, the HIV testing performed for the study in 2009 was no longer anonymous. In all surveys, results of HIV and STIs were linked with the behaviour data using a unique study number.

Finally participants were referred to a physician who offered a check-up for STIs including gynaecological examination. STIs that were detected during the examination were immediately treated free of charge. After the results of the STI tests were known, participants who needed additional treatment were contacted and invited to the health facility. These participants could be tracked via a temporary link between study number and client file. This temporary link was not created in 2007. All participants were given extra male condoms.

In all five sites, clients who were HIV positive were offered counselling by trained counsellors and psychological support through support groups for HIV positive sex workers. Another blood test was taken for a biological check-up, including CD4 count. Patients who were eligible for anti-retroviral treatment (ART) according to national guidelines received ART in the clinics for free.

### Laboratory procedures

The samples for STI testing were kept at −20°C in the local laboratories until transportation on dry ice to the STI reference laboratory of the Institute of Tropical Medicine in Antwerp, Belgium, where DNA amplification assays were performed for the detection of *C. trachomatis*, *N. gonorrhoeae* and *T. vaginalis*. *C. trachomatis* and *N. gonorrhoeae* were detected using the AMPLICOR *Chlamydia trachomatis/Neisseria gonorrhoeae* (CT/NG) test (Roche Diagnostic Systems, Indianapolis, IN) and samples testing positive were re-tested with the BD ProbeTec assay ™ ET *Chlamydia trachomatis* (CT) and *Neisseria gonorrhoeae* (GC) amplified DNA assay (Becton-Dickinson, Sparks, MD). Procedures were performed according to the manufacturer's instructions. For the detection of *T. vaginalis* the DNA was extracted using the NucliSens miniMAG (BioMérieux, Boxtel, the Netherlands). DNA templates were amplified with the TVK3/7 primer pairs [8] and positive results were confirmed using the IP1/IP2 primer pairs [9], [10]. Adequacy of the specimens and inhibition of the amplification assay were assessed by amplification of the β2-microglobulin gene. Participants were considered to be infected with *C. trachomatis*, *N. gonorrhoeae* or *T. vaginalis*, if their specimen was positive on two different molecular amplification assays or using two different primer pairs.

During the 2007 surveys the oral mucosa transudate was tested with the Oraquick HIV-1/2 Antibody Test (OraSure Technologies Inc., Bethlehem, USA) without further confirmatory testing. In 2009, blood samples obtained by finger prick were tested for HIV infection according to a HIV testing algorithm using three sequential rapid HIV assays. In a first step, Determine™ (Abbott Laboratories by Abbott Japan CO. LTD, Minato-Ku, Tokyo Japan) was applied. Specimens reactive on Determine were subsequently tested using the SD Bioline HIV-1/2 (Standard Diagnostics, Kyonggi-do, South Korea). Specimens giving discordant results were re-tested with HIV1/2 Stat-Pak (Chembio Diagnostics Systems, New York 11763, USA) as a tie-breaker.

Specimens with reactive results in two rapid assays were considered to be HIV positive.

### Data management and analysis

Data were entered in Epi-Info 3.4.3 (CDC, Atlanta, USA). All statistical analyses were done using Intercooled Stata 11.0 (Stata Corporation, Texas 77845 USA). Socio-demographic characteristics, sexual behaviour, condom use, prevalences of STIs and of HIV were compared between FSW coming for the first visit and FSW coming for a routine visit. Differences were tested on their statistical significance with the chi-square or the Fisher exact test where appropriate. In addition HIV prevalence among first visit FSWs in the survey was compared with HIV prevalence among FSW attending counselling and testing services, which are routinely offered to all FSWs on their first visit at the same clinics.

### Ethical considerations

All biological specimens and study forms, including questionnaires were labelled with a study number unlinked to the participant. No names or other identifiers were recorded. Temporary links created for tracing STI infected participants were immediately destroyed after use.

The study was approved by the National Ethical Committee of Life Sciences and Health of Côte d'Ivoire, the Institutional Review Board of the Institute of Tropical Medicine and the Ethical Committee of the University of Antwerp in Belgium, and the Protection of Human Subjects Committee of FHI.

## Results

### Socio-demographic and behavioural characteristics

A total of 1110 FSWs participated in the surveys in 2007 and 2009. Selected socio-demographic and behavioural characteristics are shown in table 1, as well as a comparison between FSW coming for the first time and FSW coming for a routine visit. The Clinique de Confiance in Marcory and the clinic in San Pedro which were open for a longer time received proportionally more women on routine visits. FSW coming for the first time were younger and more often Ivoirian. They had spent a shorter time in Côte d'Ivoire and reported more frequently a regular, non paying sexual partner. The median duration of sex work was two years, and the median number of clients during the last working day was two. Consistent condom use with clients, which was defined as the proportion of FSW who always used condoms during the last working day with their clients, was lower among women attending for the first time (66.9% vs. 77.5%, p<0.001). Consistent condom use with the regular partner was defined as the proportion of FSWs who always used condoms during the last week with their regular, non paying partner such as their husband, boyfriend. In this case condom use was much lower, 26,4%, and did not differ significantly between FSW coming for the first time and for a routine visit. The proportion who never had done a HIV test was 64.9% among FSW coming for the first time and 25.9% among FSW coming on a routine visit (p<0.001).

**Table 1:** Socio-demographic characteristics and STI/HIV prevalence among female sex workers (FSW) coming for the first time to the clinics compared with FSW coming for a routine visit.

	All FSW	FSW coming for the first time	FSW on a routine visit	P (§)
	N = 1110	N = 510	N = 600	
SW center (%)				<0.001
Abidjan, Marcory	223 (20.1)	61 (12.0)	162 (27.0)	
Abidjan, Yopougon	220 (19.8)	106 (20.8)	114 (19.0)	
San Pedro	221 (19.9)	78 (15.3)	143 (23.8)	
Yamoussoukro	225 (20.3)	152 (29.8)	114 (19.0)	
Gagnoa	221 (19.9)	113 (22.2)	108 (18.0)	
Median age, yrs (IQR)	25 (20–30)	22 (19–27)	27 (22–34)	<0.001
Country of origin (%)				<0.001
Côte d'Ivoire	922 (83,1)	459 (90.0)	463 (77.2)	
Nigeria	31 (2,8)	6 (1.2)	25 (4.2)	
Liberia	63 (5,7)	13 (2.5)	50 (8.3)	
Ghana	71 (6,4)	21 (4.1)	50 (8.3)	
Other	23 (2,1)	11 (2.2)	12 (2.0)	
Schooling level (%)				0.63
None	407 (36.7)	185 (36.3)	222 (37.0)	
Primary school	342 (30.8)	160 (31.4)	182 (30.3)	
Secondary school	331 (29.8)	148 (29.0)	183 (30.5)	
Higher	30 (2.7)	17 (3.3)	13 (2.2)	
Regular partner or boyfriend (%)	830 (74.9)	402 (79.0)	428 (71.4)	0.004
Median duration of sex work (years)	2 (1–5)	2 (0–3)	3 (2–5)	<0.001
Median nb clients last working day	2 (1–4)	2 (1–3)	2 (1–4)	0.02
Median price paid by last client (in US $)	4 (2–10)	4 (2–10)	4 (2–10)	0.73
Condom use with last client (%)	863 (77.7)	376 (73.7)	487 (81.2)	0.003
Consistent condom use with clients*(%)	779 (72.6)	332 (66.9)	447 (77.5)	<0.001
Use of lubricant gel (%)	213 (19.2)	58 (11.4)	155 (25.8)	<0.001
Consistent condom use with regular partner**(%)	125 (26.4)	52 (23.3)	73 (29.2)	0.15
Drug use (%)				0.60
None	1082 (97.5)	499 (97.8)	583 (97.2)	
Injectable	1 (0,1)	0 (0.0)	1 (0.2)	
Non injected hard drugs	1 (0,1)	0 (0.0)	1 (0.2)	
Soft drugs	26 (2.3)	11 (2.2)	15 (2.5)	
HIV test in the past (%)				<0.001
Never	486 (43.9)	331 (64.9)	155 (25.9)	
Once	326 (29.4)	99 (19.4)	227 (38.0)	
Two times	169 (15.2)	46 (9.0)	123 (20.6)	
At least three times	127 (11.5)	34 (6.7)	93 (15.5)	
*N. gonorrhoeae* (NG)	56 (5,1)	28 (5,5)	28 (4.7)	0.53
*C. trachomatis* (CT)	58 (5,2)	40 (7,9)	18 (3.0)	<0.001
*T. vaginalis* (TV)	139 (12,9)	72 (14,5)	67 (11.5)	0.15
NG or CT	101 (9,1)	60 (11,8)	41 (6.9)	0.004
NG or CT or TV	221 (20,6)	122 (24,6)	99 (17.1)	0.002
HIV	266 (26,6)	78 (17,5)	188 (33.9)	<0.001

§ Chi square or Kruskal Wallis test.

* Consistent condom use with clients during last working day.

** Consistent condom use with regular partner during last week.

Note: some of the numbers don't add up to the totals because of missing values.

### Prevalence of HIV and STI

The prevalence of STIs and HIV is also shown in table 1, for all participants, as well as the comparison first-time versus routine attendees. The prevalence of *N. gonorrhoeae* was 5%, and no difference was observed between first time and routine visits. FSW coming to the clinics for the first time had a higher prevalence of *C.trachomatis* (7.9% vs. 3.0%, p<0.001). The prevalence of HIV was 26.6%, 17.5% in first attendees and 33.9% in FSW coming for a routine visit (p<0.001).

### Comparison of HIV prevalence among survey participants and FSW attending routine VCT services”

HIV prevalence data from the surveys in 2007 and 2009 were compared with HIV results from routine counselling and testing services from the same clinic at approximately the same period and are presented in table 2. Only data from first time attendees were taken. Acceptance of the HIV test during the surveys was 87.4%, and much higher than the 43.0% during routine HIV testing and counselling services (p<0.001). The HIV prevalence obtained from the survey was lower as compared to the proportion of HIV positive results at routine counselling and testing services, although this difference did not reach statistical difference (17. 5% vs. 21.8%, p = 0.08).

**Table 2:** Prevalence of HIV as measured by the surveys and proportion of HIV positive FSW in routine counseling and testing services (*), for first time attendees and per study site.

	All	Clinique de Confiance, Abidjan	CAMES, Abidjan	Centre Espérance, San Pedro	CMSA,Yamoussoukro	CAMES,Gagnoa
STUDY RESULTS						
Study period		15/10/07–14/11/07	19/10/07–28/11/07	13/11/07–29/11/07	16/06/09–25/08/09	03/06/09–24/08/09
N (study participants visiting for first time)	510	61	106	78	152	113
N HIV tests (% accepted HIV test)	446(87,4)	61(100,0)	102(96,2)	78(100,0)	110(72,4)	95(84,1)
N HIV tests positive	78	19	15	21	12	11
HIV positive (%)	17,5	31,1	14,7	26,9	10,9	11,6
ROUTINE COUNSELING AND TESTING SERVICES						
Period		01/04/07–30/09/07	01/04/07–30/09/07	01/05/07–31/10/07	01/12/08–31/05/09	01/12/08–31/05/09
N first clinic attendances	1322	336	412	306	74	194
N HIV tests (% accepted HIV test)	569(43,0)	202(60,1)	117(28,4)	106(34,6)	31(41,9)	11358,2)
N HIV tests positive	124	43	30	32	9	10
HIV positive (%)	21,8	21,3	25,6	36,8	29,0	11,5

* 6 months previous study period.

## Discussion

The PAPO-HV project in Côte d'Ivoire has been cited as an example of how a small-scale successful targeted intervention for sex workers was translated into a large-scale public health intervention at country level [1]. Present study results show that female sex workers remain at high risk to acquire and transmit HIV and that continuous efforts and strengthening of prevention activities will be needed to have an impact on the HIV transmission dynamics in the country.

Evaluation of outcome and impact is one of the major challenges of sex worker projects. Yet, a rigorous evaluation is needed in order to provide the evidence base for an increased response to HIV. Population based surveys among a representative sample of FSWs are the best method to obtain information on behaviour characteristics and STI/HIV prevalence in the target population. However, no sampling frame exists for FSW, different alternative sampling methods have been proposed with variable success in decreasing some of the inevitable biases of non-probability sampling [11]. Furthermore, conducting a nationwide bio-behavioural survey among sex workers is logistically complicated and expensive. Furthermore, although monitoring and evaluation is considered an essential part of a FSW project or programme, the budget allocated to monitoring and evaluation is often not sufficient for conducting FSW population based bio-behavioural surveys. Repeated facility-based surveys however may be very useful to provide repeated STI and HIV data and to conduct sentinel surveillance for sex workers. If selection and participation bias remain similar over time, then trends in infection recorded in sentinel sites will reflect trends in the population represented by those sites [12]. It should be admitted that changes in selection bias are difficult to assess and can never be completely excluded. In our study for instance, ART has been made increasingly available at the clinics, which could have attracted increasingly HIV positive women to the clinics and thus a changing bias towards higher HIV prevalences. In San Pedro and in the Clinique de Confiance in Abidjan, where ART services have been offered for a longer period of time as compared with the other sites, HIV prevalence was much higher (42.0% in clinics offering ART vs 14.3% in clinics not offering ART, p<0.001). All these biases notwithstanding, clinic based surveys may still be very useful for evaluation purposes through triangulation with other data sources. By comparing different multiple datasets with each other, triangulation helps to counteract threats to the validity of each data source [13]. Previous studies at the Clinique de Confiance in Abidjan for instance have demonstrated the effectiveness of a similar intervention by monitoring behaviour and STI and HIV prevalence among female sex workers attending the clinic for the first time and comparing these results with behaviour characteristics from community based surveys [4].

In our setting, “baseline” surveys were conducted some years after the project was initiated, and one may ask whether these surveys were still relevant and needed at that time. Late baseline surveys are a problem faced by many projects for hidden and hard-to-reach target populations. It is crucial to gain access to and confidence from the target population for conducting surveys among those populations. Therefore it is not very realistic, if not impossible, to conduct a real baseline survey before initiating any activity including setting up a network of peer educators. Although “late” for the PAPO-HV project (which ended in 2010), data from this study may be used in future studies as baseline data, in order to assess trends in condom use and STI/HIV prevalence, and can be triangulated with other data sources.

Another limitation of the study is the fact that we were not able to check for independency of the study samples. Some of the FSW may have participated more than once in the study, as the study was strictly anonymous and there was a time gap between the two sets of studies.

In the surveys conducted in 2009 the results from the routine testing algorithm were used during the survey, and no separate HIV test was done, as in 2007. In this study we examined whether routine HIV counselling and testing services might be used for measuring trends in HIV prevalence, without conducting a separate survey to this purpose. Only 9,3% of the first time attendees had recently done a HIV test (results not shown), so the majority of them were eligible for a HIV test. One of the major problems however was the low acceptance of the HIV test in routine conditions. During the survey, more efforts were probably made to recruit women for HIV testing than in the routine situation. If data from routine counselling and testing services are to be used, a strategy should be developed to increase the acceptance of HIV test in the surveillance sites. As for surveillance of other STIs, the acceptance of a self-administered vaginal swab was high. Avoiding a gynaecological examination, which is time-consuming, could add to the feasibility of conducting regular surveys among female sex workers.

It was for the first time in Côte d'Ivoire that STI and HIV prevalences among sex workers have been assessed outside the Clinique de Confiance in Abidjan. San Pedro is the fourth town in Côte d'Ivoire and an important port city, with a HIV prevalence of 44.3% in clinic attendees. CAMES Abidjan, Yamoussoukro and Gagnoa showed all somewhat lower HIV prevalences, but these are still twice or three times the prevalence in the general population. In addition, condom use was very low. Although 72.6% of the FSW reported consistent condom use with clients only 26.4% did so with the regular partner. As a comparison, the proportion of FSW reporting consistent condom use with clients was already 78% at the Clinique de Confiance in 1998 [4], The low condom use and very high STI prevalence which were found in this study suggest ongoing transmission of STIs and HIV between this core group and the general population, via the clients and boyfriends.

Despite the evidence that sex workers are the priority key population to target prevention efforts in Cote d'Ivoire and that programs can be successfully scaled up and monitored, sustainability and continuation of the PAPO-HV project is a major problem. Both the recent period of political instability and the abrupt change of the technical partner by the main donor resulted in serious interruptions in the programme. These management issues are as important for the success of prevention among sex workers in Cote d'Ivoire as the technical aspects of program monitoring and evaluation.

In conclusion, our results show a relatively high STI and HIV prevalence among FSWs in different cities in Côte d'Ivoire. Prevention efforts should continue to focus on FSWs in order to have an impact on the HIV epidemic in the country.

Part of the data were presented at the 18th International Society for STD Research Conference ISSTDR 2009, London 28 June–1 July. Abstract P. 3.162.
